# Relevance of plasma biomarkers to pathologies in Alzheimer’s disease, Parkinson’s disease and frontotemporal dementia

**DOI:** 10.1038/s41598-022-22647-6

**Published:** 2022-10-26

**Authors:** Pai-Yi Chiu, Fu-Chi Yang, Ming-Jang Chiu, Wei-Che Lin, Cheng-Hsien Lu, Shieh-Yueh Yang

**Affiliations:** 1 Department of Neurology, Show Chwan Memorial Hospital, Chunghwa, 500 Taiwan; 2 MR-Guided Focus Ultrasound Center, Chang Bin Shaw Chwan Memorial Hospital, Changhwa, 505 Taiwan; 3 Department of Neurology, Tri-Service General Hospital, National Defense Medical Center, Taipei, 114 Taiwan; 4 Department of Neurology, National Taiwan University Hospital, College of Medicine, National Taiwan University, Taipei, 100 Taiwan; 5 Graduate Institute of Brain and Mind Sciences, College of Medicine, National Taiwan University, Taipei, 100 Taiwan; 6 Department of Psychology, National Taiwan University, Taipei, 106 Taiwan; 7 Graduate Institute of Biomedical Electronics and Bioinformatics, National Taiwan University, Taipei, 106 Taiwan; 8 Department of Diagnostic Radiology, Kaohsiung Chang Gung Memorial Hospital, College of Medicine, Chang Gung University, Kaohsiung, 833 Taiwan; 9 Department of Neurology, Kaohsiung Chang Gung Memorial Hospital, College of Medicine, Chang Gung University, Kaohsiung, 833 Taiwan; 10 MagQu Co., Ltd., New Taipei City, 231 Taiwan

**Keywords:** Biochemistry, Biotechnology, Neuroscience

## Abstract

Amyloid plaques and tau tangles are pathological hallmarks of Alzheimer’s disease (AD). Parkinson’s disease (PD) results from the accumulation of α-synuclein. TAR DNA-binding protein (TDP-43) and total tau protein (T-Tau) play roles in FTD pathology. All of the pathological evidence was found in the biopsy. However, it is impossible to perform stein examinations in clinical practice. Assays of biomarkers in plasma would be convenient. It would be better to investigate the combinations of various biomarkers in AD, PD and FTD. Ninety-one subjects without neurodegenerative diseases, 76 patients with amnesic mild cognitive impairment (aMCI) or AD dementia, combined as AD family, were enrolled. One hundred and nine PD patients with normal cognition (PD-NC) or dementia (PDD), combined as PD family, were enrolled. Twenty-five FTD patients were enrolled for assays of plasma amyloid β 1–40 (Aβ_1–40_), Aβ_1–42_, T-Tau, α-synuclein and TDP-43 using immunomagnetic reduction (IMR). The results show that Aβs and T-Tau are major domains in AD family. α-synuclein is highly dominant in PD family. FTD is closely associated with TDP-43 and T-Tau. The dominant plasma biomarkers in AD family, PD family and FTD are consistent with pathology. This implies that plasma biomarkers are promising for precise and differential assessments of AD, PD and FTD in clinical practice.

## Introduction

In addition to cognitive and behavioral inspections, the demand for biological examinations is strongly increasing for neurodegenerative diseases in clinics^1–3^. From the perspective of pathogenesis, neurodegenerative diseases result from the misfolding of specific proteins in the brain^4–6^. Pathological studies on animal biopsy or human autopsy demonstrate that the accumulation of these misfolded proteins causes neuronal damage, cognitive impairment and behavioral disorders^7–11^. For example, amyloid plaques and tau-protein tangles are pathological hallmarks of Alzheimer’s disease (AD)^12–14^. Lewy bodies composed of α-synuclein were found in the brain steins of patients with Parkinson’s disease (PD)^15,16^. TAR DNA-binding protein (TDP-43) and total tau protein (T-Tau) play roles in the pathology of patients with frontotemporal dementia (FTD)^17,18^. To observe the pathological evidence of neurodegenerative diseases, positron emission tomography (PET) scans with the aid of tracers to specifically label the accumulated proteins in brains have been developed or are under development^19–22^. Unfortunately, to date, only tracers for amyloid PET are available in clinical practice. It is impossible to use PET scans to clarify the pathologies for PD or FTD in clinics. Even for amyloid PET scans, the cost issue makes it difficult to perform in routine practice. Assays of these biomarkers in cerebrospinal fluid (CSF) are alternative inspections for neurodegenerative diseases^23–26^. Several research results show high correlations between CSF biomarkers and pathology or clinical diagnosis^27–31^. However, due to side effects, lumbar puncture is not popularly used in clinical practice. Thus, biological examinations with biomarkers face severe bottlenecks.

With the successful development of ultrasensitive assay technologies in the early 2000s, the precise detection of biomarkers in human plasma instead of CSF has become feasible^32–35^. Many researchers and neurologists have been interested in studies on plasma Aβ, T-Tau, TDP-43 and other biomarkers. Most studies have concentrated on the discrimination between individual neurodegenerative diseases and normal controls using plasma biomarkers. It was reported that plasma Aβ_1–42_ and T-Tau are able to differentiate AD from normal controls^36–39^. PD patients show significantly higher levels of plasma α-synuclein than normal controls^40,41^. Relatively higher levels of plasma TDP-43 in FTD were reported^42^. However, comprehensive studies on the effects of Aβs, T-Tau, α-synuclein and TDP-43 on AD, PD and FTD are rare.

In this work, normal controls and patients with either AD, PD or FTD were enrolled. Plasma Aβ_1–40_, Aβ_1–42_, T-Tau, α-synuclein and TDP-43 were assayed for each participant using immunomagnetic reduction (IMR). Since 2011, the results of assaying plasma Aβ_1–40_, Aβ_1–42_, T-Tau, α-synuclein and TDP-43 have been reported by independent groups over the world^36,37,40–43^. In addition to ultra-high sensitivity and specificity, the published results evidenced the high consistencies in measured plasma biomarkers with CSF biomarkers^44^, plasma Aβ_1–42_/Aβ_1–40_ with amyloid PET^45^, plasma T-Tau with magnetic resonance MRI^46^, plasma Aβ_1–42_ xT-Tau, α-synuclein, and TDP-43 with clinical diagnosis of AD, PD and FTD, respectively^37,42,47–49^. With these clinical validations, IMR kits have been registered with CE IVD and approved as an in-vitro medical devices by Taiwan Food and Drug Administration. Hence, IMR is reliable to be used to explore the plasma Aβ_1–40_, Aβ_1–42_, T-Tau, α-synuclein and TDP-43. The contributions of each plasma biomarker to AD, PD and FTD were analyzed to clarify the role of each biomarker in these neurodegenerative diseases.

## Methods

### Enrollment of subjects

All subjects were enrolled at hospitals in Taiwan with approvals by the ethics committees of every hospital. Each subject was identified as normal control (NC), Alzheimer’s disease (AD), Parkinson’s disease (PD) or frontotemporal dementia (FTD) according to the diagnostic guidelines of NIA-AA^50^, United Kingdom PD Society Brain Bank^51^, and frontotemporal lobe degeneration^52^. AD patients were diagnosed with neuropsychological tests such as clinical dementia rating (CDR), mini-mental state examination (MMSE), activities of daily living scale, instrumental activities of daily living scale, etc. AD patients having MMSE scores between 24 and 28 and CDR = 0.5 are amnesic mild cognitive impairment, having MMSE scores between 10 and 22 and CDR = 0.5, 1 or 2 are AD dementia. PD patients must have symptoms of bradykinesia and at least one of muscular rigidity, rest tremor (4–6 Hz), or postural instability unrelated to primary visual, cerebellar, vestibular or proprioceptive dysfunction In addition, PD patients have three or more of unilateral onset, resting tremor, progressive disorder, persistent asymmetry most affecting the side of onset, excellent response to levodopa, severe levodopa-induced chorea, levodopa response for over 5 years, and clinical course of over 10 years. Examinations of CRD, MMSE and Hoehn–Yahr (H–Y) stage were performed for PD patients. PD patients having CDR = 0 are PD with normal cognition. PD patients having MMSE scores between 10 and 26 and CDR = 0.5, 1 or 2 are PD dementia FTD patients were diagnosed with mainly primary progressive aphasia. Subjects with cranial metallic implants, cardiac pacemakers or claustrophobia, significant history of depression, and geriatric depression scale > 8, history of repeated strokes with stepwise progression and repeated head injury were excluded. Four experienced neurologists were involved in the diagnostic process. All enrolled subjects were examined with brain magnetic resonance imaging. All experiments were performed in accordance with relevant guidelines and regulations. All participants provided written informed consent prior to study enrollment.

### Preparation of plasma samples

A 9 ml or 6 ml K3 EDTA lavender-top tube was used for blood draw with each enrolled subject, followed by centrifugation at 1500–2500 g at room temperature for 15 min. Plasma was collected and aliquoted into cryotubes (0.5 ml aliquots) and stored at −20 °C. The freezing of plasma should be performed within 4 h after blood draw. Each frozen plasma sample was placed in wet ice and then positioned at room temperature for IMR measurement.

### Assays of biomarkers

IMR reagents (MF-AB0-0060, MF-AB2-0060, MF-TAU-0060, MF-ASC-0060, MF-TDP-0060, MagQu) were used to assay Aβ_1–40_, Aβ_1–42_, T-Tau, α-synuclein and TDP-43 in plasma with the aid of an IMR analyzer (XacPro-S, MagQu). There are two stages of quality controls for each batch of IMR measurement. The first stage to is calibrate the reading of the sensor of the analyzer. 120-μl, 10-mg-Fe/ml magnetic fluid is used as a calibrator for each detecting channel. The output signals of blank test (without calibrator) and calibrator test (with calibrator) should be within the acceptable range. In addition, the signal ratio of calibrator test to blank test should be higher than 20. The second stage is as follows. For each batch of IMR measurement for a given biomarker, control samples (several tens of pg/ml) with known concentrations are assayed together with tested samples. The deviations of measured concentrations of control samples should be less than 15%. Duplicated measurements were performed for each biomarker of a plasma sample. The averaged value of the duplicated measurements was reported.

### Statistical method

The software, GraphPad Prism 6.01, was used to perform the data analysis. Ages, CDR, MMSE scores, H–Y stage, concentrations of biomarkers were presented as the means ± standard deviations for each enrolled group. MMSE scores and concentrations of biomarkers were compared between two enrolled groups, e.g. AD family versus NC, using a t test, and *p* values were determined. The value of 0.05 for *p* was a criterion to determine the significance.

### Ethical standards

The study was started and conducted after approval of the study protocol by the regional ethical committee in all the joined hospitals, including Chang Bin Show Chwan Memorial Hospital, Tri-Service General Hospital, National Taiwan University Hospital, and Kaohsiung Chang Gung Memorial Hospital. The study was carried out in accordance with relevant guidelines and regulations, including the World Medical Association (WMA) Declaration of Helsinki. A written informed consent was obtained from all participants and/or their legal guardians.

## Results

The demographic information of the enrolled subjects is listed in Table 1. Ninety-one normal controls aged 64.1 ± 6.8 years were enrolled and referred to as the NC group. The female percentage was 65.9%. The clinical dementia ranking (CDR) of every participant in the NC group was zero. The score of the mini-mental state examination (MMSE) was 28.6 ± 1.6. The AD family had forty-one patients with amnesic cognitive impairment and thirty-five patients with AD dementia. The female percentage was 72.4%. The age of the AD family was 74.0 ± 9.8 years. The CDR of the AD family was 0.78 ± 0.52, and the MMSE score was 23.1 ± 5.3, which were significantly lower than those of the NC group (*p* < 0.0001). The PD family had 47 PD patients with normal cognition and 62 patients with PD dementia. The female percentage was 67.9%. The age of the PD family wad 65.8 ± 10.4 years. The CDR was 0.40 ± 0.47. The MMSE score was 22.4 ± 5.7, which resulted in *p* < 0.0001 compared to the NC group. The Hoehn and Yahr (H–Y) stage of the PD family was 2.20 ± 1.1. The FTD group had 25 patients with frontotemporal dementia. The female percentage was 60.2%. The age of the FTD group was 75.2 ± 11.6 years. The CDR and MMSE scores were not available for the FTD group.

**Table 1:** Demographic information and measured concentrations of plasma biomarkers in enrolled subjects.

Group	NC	AD family	PD family	FTD
n (female%)	91 (65.9%)	76 (72.4%)	109 (67.9%)	25 (60.2%)
Age (years)	64.1 ± 6.8	74.0 ± 9.8	65.8 ± 10.4	75.2 ± 11.6
CDR	0	0.78 ± 0.52	0.40 ± 0.47	–
MMSE	28.6 ± 1.6	23.1 ± 5.3****	22.4 ± 5.7****	–
H–Y stage	–	–	2.20 ± 1.1	–
Aβ_1-40_ (pg/ml)	58.68 ± 13.28	49.70 ± 15.58****	44.96 ± 10.88****	40.34 ± 4.69***
Aβ_1-42_ (pg/ml)	15.72 ± 2.48	19.61 ± 5.21****	17.08 ± 3.37**	18.42 ± 3.02****
T-Tau (pg/ml)	17.46 ± 9.28	34.51 ± 9.91****	26.64 ± 10.36****	41.53 ± 20.51****
α-synuclein (fg/ml)	96.7 ± 179.8	414.8 ± 1345*	3648 ± 9065**	54.3 ± 59.7
TPD-43 (pg/ml)	0.165 ± 0.062	0.154 ± 0.106	0.116 ± 0.075	0.356 ± 0.202****

*NC* normal control, AD family patients with amnestic cognitive impairment or Alzheimer’s disease, *PD family* Parkinson’s disease patients with or without dementia, *FTD* frontotemporal dementia, *CDR* clinical dementia ranking, *MMSE* mini-mental state examination, *H–Y stage* Hoehn and Yahr stage.

*p < 0.05 using NC as reference.

**p < 0.01 using NC as reference.

***p < 0.001 using NC as reference.

****p < 0.0001 using NC as reference.

The measured levels of plasma biomarkers in the NC, AD family, PD family and FTD groups are listed in Table 1. According to published papers^49,53,54^, the concentrations of plasma biomarkers assayed with immunomagnetic reduction (IMR) in normal controls are independent of age. Hence, it is not necessary to have corrections to age in the analysis. For plasma Aβ_1–40_, the NC group showed significantly higher levels (58.68 ± 13.28 pg/ml) than the AD family (49.70 ± 15.58 pg/ml; *p* < 0.0001), PD family (44.96 ± 10.88 pg/ml; *p* < 0.0001) and FTD (40.34 ± 4.69 pg/ml; *p* < 0.001). However, NC showed significantly lower levels of plasma Aβ_1–42_ (15.72 ± 2.48 pg/ml) than the AD family (19.61 ± 5.21 pg/ml; *p* < 0.0001), PD family (17.08 ± 3.37 pg/ml; *p* < 0.01) and FTD (18.42 ± 3.02 pg/ml; *p* < 0.0001).

Significantly higher levels of plasma T-Tau were found in the AD family (34.51 ± 9.91 pg/ml; *p* < 0.0001), PD family (26.64 ± 10.36 pg/ml; *p* < 0.0001) and FTD (41.53 ± 20.51 pg/ml; *p* < 0.0001) than in the NC group (17.46 ± 9.28). Remarkably, FTD showed relatively high levels of plasma T-Tau compared with the other groups. However, FTD showed equivalent levels of plasma α-synuclein (54.3 ± 59.7 fg/ml) to the NC group (96.7 ± 179.8 fg/ml). The AD and PD families showed higher levels of plasma α-synuclein (AD: 414.8 ± 1345 fg/ml; *p* < 0.05, PD: 3648 ± 9065 fg/ml, *p* < 0.01) than the FTD and NC groups. Notably, the PD family had much higher levels of α-synuclein than the AD family.

Relatively higher levels of plasma TDP-43 were found in the FTD (0.356 ± 0.202 pg/ml, *p* < 0.0001) than in the NC group (0.165 ± 0.062 pg/ml), AD family (0.154 ± 0.106 pg/ml) and PD family (0.116 ± 0.075 pg/ml). There was no significant difference in plasma TDP-43 levels among the NC group, AD family and PD family.

The effect sizes of each biomarker in the AD family, PD family and FTD group were investigated. For a biomarker, the effect size is calculated via the ratio of the mean concentration in a given neurodegenerative disease to that in the NC group. For example, the effect size of T-Tau for the AD family was calculated as 34.51/17.46 = 1.98. The levels of plasma Aβ_1–40_ in the AD family, PD family and FTD group decreased compared to the NC group. Instead of Aβ_1–40_, the inversion of Aβ_1–40_, i.e., 1/Aβ_1–40_, was used to calculate the effect size.

The effect sizes of biomarkers for all groups are listed in Table 2. For 1/Aβ_1–40_, the effect size ranged from 1.18 to 1.45 among the neurodegenerative diseases. For Aβ_1–42_, the AD family showed the highest value (= 1.25) for the effect size. This result demonstrates the role of amyloid β in AD. The FTD group showed the highest value of effect size in plasma T-Tau (= 2.38). In addition to T-Tau, the FTD group showed a relatively higher value (= 2.15) for the effect size in plasma TDP-43. The results revealed that the average levels of plasma T-Tau and TDP-43 in the FTD group were more than double those in the NC group. The average level of plasma T-Tau in the AD family was almost double that in the NC group. The PD family showed a relatively high value of effect size in plasma α-synuclein (= 37.7).

**Table 2:** Effect sizes of measured plasma biomarkers in subjects with neurodegenerative diseases.

Group	AD family	PD family	FTD
1/Aβ_1–40_ (ml/pg)	1.18	1.31	1.45
Aβ_1–42_ (pg/ml)	1.25	1.09	1.17
T-Tau (pg/ml)	1.98	1.53	2.38
α-Synuclein (fg/ml)	4.29	37.7	0.56
TPD-43 (pg/ml)	0.93	0.70	2.15

*AD family* patients with amnestic cognitive impairment or Alzheimer’s disease, *PD family* Parkinson’s disease patients with or without dementia.

Notably, there are two reasons for this issue. Firstly, the levels of other biomarkers such as Aβ_1–42_, T-Tau, α-synuclein and TDP-43 elevate in AD, PD or FTD as compared to NC. The effect sizes of these biomarkers are higher than 1. However, the level of Aβ_1–40_ decreases in dementia groups as compared to NC. The effect size should be lower than 1, which is not consistent with other biomarkers. In order to have common expressions to show the increases in biomarker levels in dementia, the inversion of Aβ_1–40_, i.e., 1/Aβ_1–40_, is used to calculate the effect size.

Secondly, if Aβ_1–40_ is used for calculating the effect size, the more changes in Aβ_1–40_ in AD, PD or FTD result in the lower values of the effect size. However, for other biomarkers, the more changes result in higher values of the effect size. This would under-estimate the dominance (or normalized effect size) of Aβ_1–40_ among biomarkers for AD, PD and FTD, as listed in Table 3. Hence, the inversion of Aβ_1–40_, i.e., 1/Aβ_1–40_, is used to calculate the effect size to avoid this paralogism.

**Table 3:** Normalized effect sizes of measured plasma biomarkers in subjects with neurodegenerative diseases.

Group	AD family	PD family	FTD
1/Aβ_1–40_ (ml/pg)	0.300	0.331	0.369
Aβ_1–42_ (pg/ml)	0.356	0.310	0.334
T-Tau (pg/ml)	0.336	0.259	0.405
α-Synuclein (fg/ml)	0.101	0.886	0.013
TPD-43 (pg/ml)	0.246	0.186	0.568

*AD family* patients with amnestic cognitive impairment or Alzheimer’s disease, *PD family* Parkinson’s disease patients with or without dementia.

## Discussion

As listed in Table 2, specific biomarkers show effect sizes of relatively higher values in AD, PD or FTD. It is worth investigating the dominance of biomarkers in neurodegenerative diseases (NDDs). In Table 2, the ranges of effect sizes among biomarkers differ greatly from each other. For example, the effect size of plasma α-synuclein ranges from 0.65 to 37.7, whereas it ranges from 1.09 to 1.25 for plasma Aβ_1–42_. To eliminate the difference in the ranges of effect size among biomarkers, the effect size of a given biomarker is normalized to the sum of effect sizes over NDDs. For instance, the normalized effect size of plasma Aβ_1–42_ in the AD family was calculated as 1.25/(1.25 + 1.09 + 1.17) = 0.356. The normalized effect sizes of every biomarker in the AD family, PD family and FTD group are listed in Table 3.

**Figure 1:**
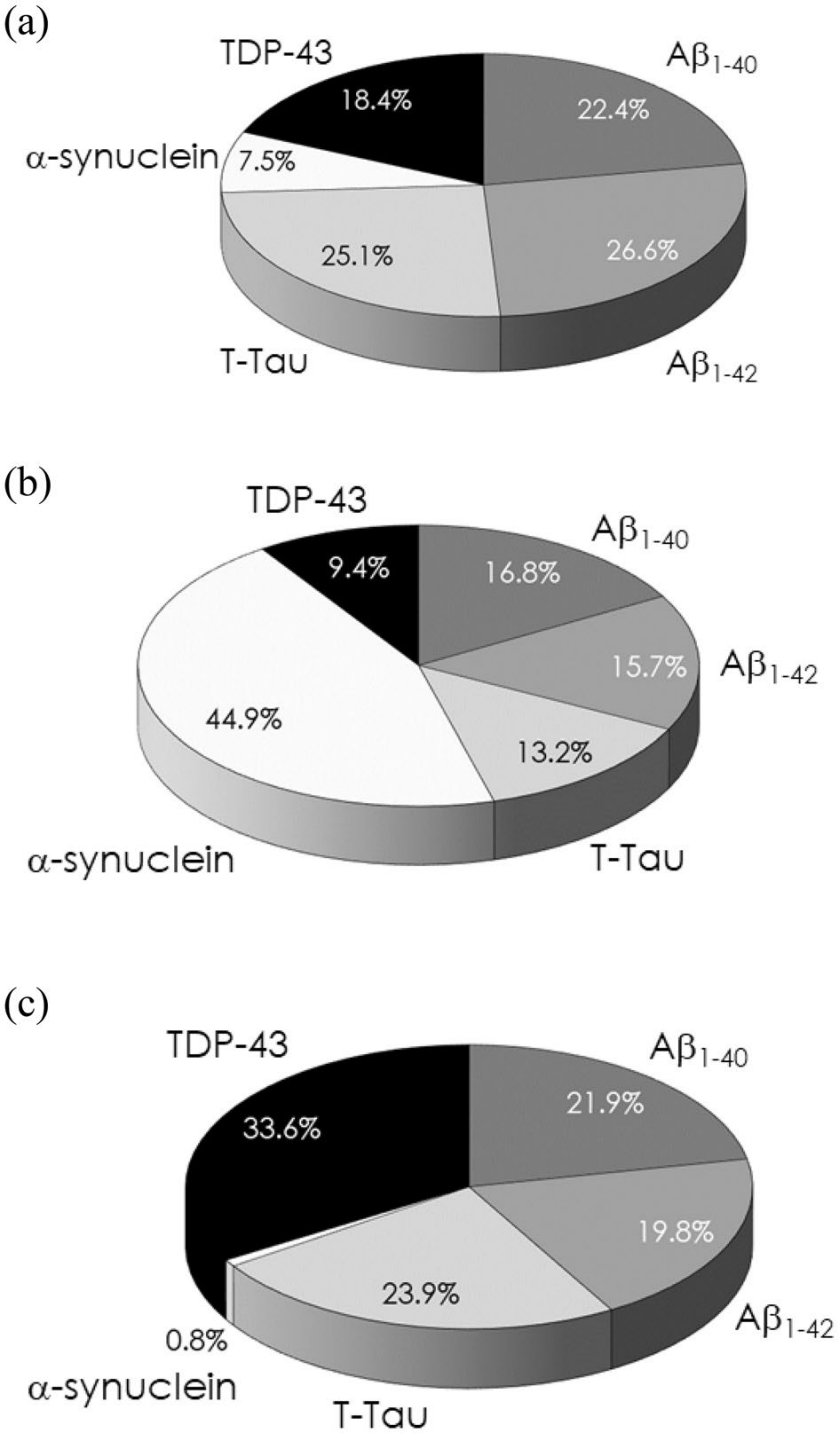
Dominance of plasma Aβ_1–40_, Aβ_1–42_, T-Tau, α-synuclein and TDP-43 in the (**a**) AD family, (**b**) PD family and (**c**) FTD.

The dominance of a biomarker in an NDD is evaluated by calculating the percentage of the normalized effect size of the biomarkers in that of all biomarkers. According to Table 3, the dominance of plasma Aβ_1–42_ in the AD family is 0.356/(0.300 + 0.356 + 0.336 + 0.101 + 0.246) × 100% = 26.6%. The combinations of plasma Aβ_1–40_, Aβ_1–42_, T-Tau, α-synuclein and TDP-43 in the AD family, PD family and FTD are shown in Fig. 1.

For the AD family, the major domains were Aβ_1–42_ (26.6%), T-Tau (25.1%) and Aβ_1–40_ (22.4%). The three biomarkers are almost equally dominant in the AD family. This evidence highlights the associations between amyloidosis and tauopathy with AD from the plasma biomarker point of view.

It is well known that Aβs are peptides of amino acids 36–43 that result from cleavage of amyloid precursor protein (APP) by β and γ secretases^55^. Certain misfolded Aβ, such as pyroglutamate Aβ, induce the accumulation of Aβ and the formation of insoluble Aβ plaques in the brain^56,57^. The toxic Aβ plaques damage neurons, particularly those surrounding the hippocampus, resulting in memory disorders and cognitive decline in AD patients^58^.

In addition, the hyperphosphorylation of tau proteins, which are abundant in brain neurons and stabilize the microtubules of neurons, causes the death of neurons in the brain^59,60^. Due to neuron death, tau proteins are expressed by neurons, and neurofibrillary tangles can be observed in the biopsy. Meanwhile, regional atrophy of the brain occurs^46^. Therefore, brain atrophy, Aβ plaques and neurofibrillary tangles in the brain are clinical features of AD.

The results in Fig. 1a showing the major dominance of Aβ_1–42_, T-Tau and Aβ_1–40_ in the AD family is consistent with AD neuropathology. Some published papers point out that the plasma Aβ_1–42_-to-Aβ_1–40_ ratio is significantly correlated with the density of Aβ plaques in the brain in AD^45,61,62^. The elevations in plasma T-Tau levels due to regional atrophy of the brain in AD were demonstrated^46^. All the results demonstrate the feasibility of using plasma Aβs and T-Tau to assess neuropathology or brain volumetry in AD.

Remarkably, as shown in Fig. 1a, TDP-43 is a minor biomarker in AD family (18.4%). Some papers have reported that TDP-43 pathology is frequently found in AD, especially in severe AD^63–65^. AD patients with TDP-43 pathology have a rapid decline in cognition^66^. Furthermore, TDP-43 species can coexist with Tau tangles in AD^67^. All the findings provide strong evidence that TPD-43 could play a role in AD, as observed in Fig. 1a.

For the PD family shown in Fig. 1b, the most important dominant biomarker is α-synuclein, whose dominance (44.9%) is more than double that of individual Aβ_1–40_ (16.8%), Aβ_1–42_ (15.7%) and T-Tau (13.2%).

According to the pathogenesis associated with PD, as α-synuclein molecules are phosphorylated, the β-sheets and oligomerization or fibrils of α-synuclein are easily formed, followed by the formation of Lewy bodies in dopaminergic neurons^68–70^. Dopaminergic neurons with Lewy bodies become degenerative and unable to express dopamine. With the lack of dopamine, neurons in the motor cortex of a brain are damaged, which results in movement disorders in PD patients^71–73^. The formation of Lewy bodies mainly consisting of α-synuclein in PD patients was demonstrated in autopsy steins^74^. Therefore, α-synuclein is the most recognized biomarker for PD. The current results of plasma biomarkers also support the key role of α-synuclein in PD.

Using IMR assays for plasma α-synuclein, research groups have validated the high discrimination of the PD family from NC (sensitivity > 80%, specificity > 80%)^40,41,49^. Furthermore, the levels of plasma α-synuclein positively correlated with the severity of cognitive impairment^40^. The finding of the association of plasma α-synuclein levels with the thinning of the limbic cortex could support the positive correlation between plasma α-synuclein levels and cognitive decline^75^. These results reveal the importance of assaying plasma α-synuclein to assess PD in clinical practice.

In Fig. 1c, it is clear that TDP-43 is the crucial biomarker for FTD (33.6%). Since the discovery of TDP-43 in 2006, it has been found that almost 50% of FTD and amyotrophic lateral sclerosis (ALS) have TDP-43 pathology^76,77^. TDP-43 not only plays a role in nuclear transcription in relation to alternative splicing or exon skipping but also a role in RNA transport granules and in regulating local translation at distal locations^78,79^. The aggregation of TDP-43 in the cytoplasm in FTD and ALS mainly results from hyperphosphorylation, ubiquitination or C-terminal truncation of TDP-43^80–82^. Thus, TDP-43 is recognized as a biomarker for FTD and ALS. The results of plasma biomarkers in Fig. 1c present the role of TDP-43 in FTD compared to amyloid, T-Tau and α-synuclein. Notably, the results in Fig. 1c show that T-Tau is the second major biomarker in FTD.

According to reported studies, in addition to TDP-43, tauopathy is a common characteristic pathological hallmark in FTD patients^83–85^. The positive tauopathy in FTD patients was found to be due to mutations in the gene encoding tau (MAPT) on chromosome 17^86^. The mutations disrupt the normal binding of tau protein to tubulin, resulting in neuronal damage and pathological deposits of tau in the brain in FTD.

Other studies on plasma biomarkers in FTD reported increases in the levels of both TDP-43 and T-Tau compared to NC^42,87^. Especially for TDP-43, FTD patients show significantly higher levels of plasma TDP-43 than AD and PD patients^42^. These results imply that the assay of plasma TDP-43 is promising for assessing FTD in the clinic. Both TDP-43 and T-Tau should be taken into account for developing drug therapy.

In Fig. 1, amyloid β and total tau protein are equally dominant in the AD family. In the PD family, only α-synuclein is dominant. TDP-43 is the first major biomarker, while T-Tau is the second major biomarker in FTD. The results clearly reveal that more than one biomarker should be considered in AD family and FTD. Combined therapies against amyloid β and total tau protein (or phosphorylated tau protein) in AD family and TDP-43 and T-Tau (or phosphorylated tau protein) in FTD should be investigated.

AD family is split to aMCI and AD dementia. PD family is split to PD with normal cognition (PD-NC) and PD dementia. The dominance of plasma Aβ_1–40_, Aβ_1–42_, T-Tau, α-synuclein and TDP-43 in aMCI, AD dementia, PD-NC, PD dementia and FTD is analyzed, as listed in Table 4. The results are compared to Fig. 1 for AD family, PD family and FTD.

**Table 4:** Dominance of plasma Aβ_1–40_, Aβ_1–42_, T-Tau, α-synuclein and TDP-43 in aMCI, ADD, PD-NC, PDD and FTD.

Group	Aβ_1–40_	Aβ_1–42_	T-Tau	α-Synuclein	TDP-43	Group	Aβ_1–40_	Aβ_1–42_	T-Tau	α-Synuclein	TDP-43
aMCI (n = 41)	24.9%	27.3%	25.7%	4.0%	18.1%	AD family (n = 76)	22.4%	26.6%	25.1%	7.5%	18.4%
AD dementia (n = 35)	19.6%	24.2%	24.6%	8.6%	23.0%						
PD-NC (n = 47)	24.6%	22.1%	20.7%	24.0%	8.6%	PD family (n = 109)	16.8%	15.7%	13.2%	44.9%	9.4%
PD dementia (n = 62)	14.0%	13.0%	10.8%	48.7%	13.4%						
FTD (n = 25)	21.0%	18.6%	23.3%	0.7%	36.4%	FTD (n = 25)	21.9%	19.8%	23.9%	0.8%	33.6%

*aMCI* amnesic mild cognitive impairment, *AD* Alzheimer’s disease, *PD* Parkinson’s disease, *PD-NC* Parkinson’s disease with normal cognition, *FTD* frontotemporal dementia.

As AD family is split to aMCI and AD dementia, Aβ_1–40_, Aβ_1–42_ and T-Tau are dominant in aMCI, which is consistent with that in AD family. In AD dementia, the role of Aβ_1–40_ is suppressed, whereas TDP-43 becomes one of dominant biomarkers. As reported^56–58,60^, TDP-43 pathology is frequently found in AD dementia, especially in severe AD. These findings provide strong evidence that TPD-43 could play a role in AD dementia.

As PD family is split to PD-NC and PD dementia, α-synuclein remains the definitely dominant biomarker in PD dementia. However, in PD-NC, in addition to α-synuclein, other biomarkers such as Aβ_1–40_, Aβ_1–42_ and T-Tau are equally dominant. The results reveal the involvements of amyloidosis and tauopathy in PD patients with normal cognition.

Some postmortem studies show the significant existence of amyloid fibrils and neurofibrillary tangles (NFTs) in brain of PD patients^88–90^. It was suggested that aggregation of NFTs, the abnormal hyperphosphorylation of tau protein, the interaction between T-Tau and α-synuclein may all result in poor axonal transport or the cell death observed in PD patients^90^. Furthermore, the amyloidosis could contribute to the rapid progression of dementia in PD patients^91,92^. However, the uptake of ^11^C Pittsburgh compound-B was found to be normal for the PD dementia^93^. All the results imply that amyloidosis and Tauopathy have roles in early-stage PD.

## Conclusion

By using IMR assays for plasma Aβ_1–40_, Aβ_1–42_, T-Tau, α-synuclein and TDP-43 in NC, the AD family, the PD family and FTD, the dominant roles of these biomarkers in these neurodegenerative diseases were clarified. Consistent with neuropathological hallmarks, the current results show that plasma Aβs and T-Tau are major biomarkers in the AD family, while plasma TDP-43 could play a role in AD dementia. Plasma α-synuclein is dominant in the PD family compared to other biomarkers. Worth noting, amyloidosis and tauopathy also significantly contribute in early-stage PD. Plasma TDP-43 is very specific to FTD and is also involved in tauopathy. Thus, plasma biomarkers assayed with IMR clearly reflect the pathogenesis of neurodegenerative diseases. The results demonstrate the feasibility of precise and differential assessments of AD, PD and FTD using plasma biomarkers. It is strongly suggested that combined treatments against various biomarkers would be necessary for the therapy of neurodegenerative diseases.

## Data Availability

The dataset generated and analyzed in the current study is available from the corresponding author on reasonable request.
